# Detection and characterization of Hepatitis B virus double-stranded linear DNA-derived covalently closed circular DNA in chronic hepatitis B patients

**DOI:** 10.1371/journal.ppat.1013999

**Published:** 2026-02-24

**Authors:** Hsin-Ni Liu, Elena Kim, Ning Sun, Zhili Wang, ThiThuyTu Nguyen, Fwu-Shan Shieh, Yuanjie Liu, Marc G. Ghany, Raymond T. Chung, Richard K. Sterling, Selena Y. Lin, Haitao Guo, Daryl T. Y. Lau, Ying-Hsiu Su

**Affiliations:** 1 The Baruch S. Blumberg Institute, Translational Medical Science, Doylestown, Pennsylvania, United States of America; 2 Department of Microbiology and Molecular Genetics; Cancer Virology Program, UPMC Hillman Cancer Center, University of Pittsburgh School of Medicine, Pittsburgh, Pennsylvania, United States of America; 3 JBS Science Inc, Doylestown, Pennsylvania, United States of America; 4 National Institutes of Health, Liver Diseases Branch, NIDDK, Bethesda, Maryland, United States of America; 5 Massachusetts General Hospital, Harvard Medical School, Liver Center, GI Division, Boston, Massachusetts, United States of America; 6 Virginia Commonwealth University, Gastroenterology and Hepatology, Richmond, Virginia, United States of America; 7 Liver Center, Beth Israel Deaconess Medical Center, Harvard Medical School, Boston, Massachusetts, United States of America; Pennsylvania State University College of Medicine: Penn State College of Medicine, UNITED STATES OF AMERICA

## Abstract

**Background and aims:**

Hepatitis B virus (HBV) replication generates a double-stranded linear DNA (dslDNA) byproduct. This dslDNA can undergo intermolecular and intramolecular nonhomologous end-joining (NHEJ) recombination, resulting in viral integration and dslDNA-derived covalently closed circular DNAs (dsl-cccDNAs), respectively. The insertions and deletions (INDELs) at the end-joining site have been used to differentiate dsl-cccDNA from the authentic cccDNA. The prevalence and characteristics of dsl-cccDNA in chronic hepatitis B (CHB) patients remain unclear.

**Approach and results:**

HBV-targeted next-generation sequencing (NGS) was used to identify 32 dsl-cccDNA-positive candidates, 22 HBeAg(+) and 10 HBeAg(-), from 56 liver biopsies of antiviral treatment-naïve CHB patients for dsl-cccDNA confirmation and characterization by PSAD-cccDNA PCR NGS. INDELs within the DR2–1 region (nt 1600–1840) of the cccDNA were analyzed. Various clonally expanded, heterogenous ~22-nt deletions in the X gene region around nt 1760 were discovered in all 32 samples. The dsl-cccDNA species were then defined and characterized by the INDELs clustered at the DR1 surrounding region (nt 1800–1840). The proportion of dsl-cccDNA in total cccDNA was higher among HBeAg(+) compared to HBeAg(-) samples. The diversity of dsl-cccDNA species positively correlated with cccDNA levels and serum viral load, and was higher in HBeAg(+) CHB.

**Conclusions:**

dsl-cccDNA is more abundant and diverse among the HBeAg(+) CHB subjects. The existence of replication-defective dsl-cccDNA may facilitate immune evasion and HBV integration, and complicate HBV pathogenesis.

## Author summary

Hepatitis B virus (HBV) double-stranded linear DNA-derived covalently closed circular DNA (dsl-cccDNA) is generated during viral replication through the nonhomologous end-joining (NHEJ) self-ligation pathway. This process often introduces insertions and deletions (INDELs) near the joint site, distinguishing dsl-cccDNA from authentic cccDNA. While most dsl-cccDNA species are replication-defective, some remain functional and may contribute to viral antigen expression, immune evasion, and HBV integration, complicating HBV pathogenesis. In this study, we confirmed the presence and abundance of dsl-cccDNA in the livers of 32 treatment-naïve chronic HBV (CHB) patients [22 HBeAg(+) and 10 HBeAg(-)] and characterized INDEL patterns by positions and lengths. We observed high INDEL heterogeneity near DR1, a defining feature of dsl-cccDNA, with greater dsl-cccDNA abundance and diversity in HBeAg(+) patients compared to HBeAg(-) patients. Additionally, we discovered a distinct 22-nt deletion mutant in the X gene region in cccDNA population. This is the first study to characterize dsl-cccDNA in liver samples from both HBeAg(+) and HBeAg(-) CHB patients. Our discoveries warrant further investigation into the potential impact of dsl-cccDNA on HBV pathogenesis and therapeutic outcomes.

## Introduction

**Fig 1:**
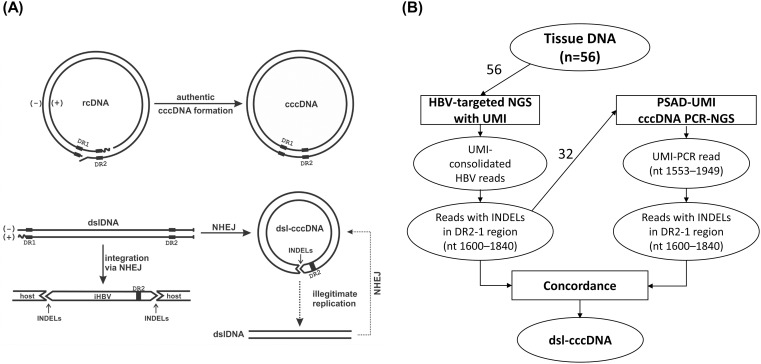
Study Design. (A) Schematic illustration of the cccDNA formation from rcDNA and dslDNA, dslDNA integration, and dsl-cccDNA-based illigimate replication (see text for details). (B) The study outline. Tissue DNA from 56 patients was subjected to HBV-targeted NGS with UMI incorporated. HBV-derived reads were extracted and analyzed for insertions and deletions (INDELs) in the DR2-1 region, as described in the Materials and Methods. Samples containing more than five distinct deletions, each supported by fewer than five UMI-consolidated reads, were considered “dsl-cccDNA positive” by HBV-targeted NGS assay. A total of 32 samples, including 30 dsl-cccDNA-positive and 2 dsl-cccDNA-negative samples as determined by HBV-targeted NGS assay, were selected for further analysis. PCR products from these samples generated by PSAD-cccDNA PCR assay were subject to PSAD-cccDNA PCR NGS. HBV-derived reads were extracted and analyzed for INDELs in the DR2-1 region. INDELs concordantly detected by both independent NGS assays were identified as originating from dsl-cccDNA and subsequently characterized in detail.

Human hepatitis B virus (HBV), an orthotype of hepadnaviruses, is the major cause of viral hepatitis, cirrhosis, and hepatocellular carcinoma (HCC) [1]. Upon infection of hepatocytes, the initiation of hepadnaviral DNA synthesis begins with the conversion of genomic relaxed circular DNA (rcDNA) of the infecting virus to covalently closed circular DNA (cccDNA) in the nucleus of the infected cell [2]. Hepadnaviruses are known to utilize sophisticated host repair mechanism(s) to repair both minus and plus-strands to form most of the cccDNA from rcDNA (Fig 1A) [3–5]. This rcDNA-derived cccDNA, known as authentic cccDNA, serves as the template for all HBV transcripts, including the pregenomic RNA (pgRNA), which acts as the replication template and supports viral persistence. However, a small fraction of cccDNA can be formed *via* self-ligation of the viral double-stranded linear DNA (dslDNA), a byproduct of reverse transcription resulting from aberrant priming of the second-strand DNA synthesis at the direct repeat 1 (DR1) instead of DR2 (Fig 1A) [3,6]. Similar to the integration of dslDNA into the host genome, the episomal self-ligation process of dslDNA is also mediated by the cellular error-prone non-homologues end-joining (NHEJ) pathway, which frequently introduces insertions or deletions (INDELs) of varying lengths at the joint site of the resulting dslDNA-derived cccDNA (dsl-cccDNA) (Fig 1A) [3,7,8]. Thus, hepadnaviral dsl-cccDNA species are commonly characterized by extensive and heterogenous INDELs at their joint site [7–12].

Due to the presence of INDELs, dsl-cccDNA species are mostly defective in supporting new rounds of rcDNA replication. However, some may remain functional for viral DNA synthesis through multiple generations of dslDNA and dsl-cccDNA intermediates—a process called illegitimate replication by Yang and Summers (Fig 1A) [9,10,12]. Additionally, some dsl-cccDNA may support viral antigen expression, similar to authentic cccDNA. Although the existence of hepadnaviral dsl-cccDNA has been demonstrated in cell cultures and in duck and woodchuck animal models [7–12], its prevalence, abundance, and function have not yet been investigated in patients with chronic hepatitis B (CHB). In this study, we sought to determine whether dsl-cccDNA species exist in CHB patients and to assess their relationship with other HBV parameters. Interestingly, we detected dsl-cccDNA in all 32 patients analyzed by next-generation sequencing (NGS). Its abundance is significantly higher in patients with HBeAg(+) CHB, independent of their viremia levels. This study is the first to detect and characterize the differential profiles of dsl-cccDNA in liver tissue from HBeAg(+) and (-) CHB patients. We also discovered a highly prevalent novel 22-nt deletion (nt 1755–1776) in the HBx gene as part of the cccDNA population. The implications of these findings for HBV pathogenesis are discussed*.*

## Results

### Detection of dsl-cccDNA by concordance analysis between HBV-targeted NGS and PSAD-cccDNA PCR NGS assays

To detect dsl-cccDNA in liver biopsies from CHB patients, this study employed two NGS approaches, specifically the HBV-targeted hybridization capture (HBV-targeted) and cccDNA PCR-based (PSAD-cccDNA PCR) NGS, as outlined in Fig 1B and detailed in Materials and Methods. HBV sequences obtained from HBV-targeted NGS may originate from all forms of HBV DNA present in each biopsy, including cccDNA, integrated HBV DNA (iDNA), and various replicative intermediates. In contrast, HBV sequences obtained from PSAD-cccDNA PCR NGS are derived speficifically from DNA templates resistant to heat denaturation and PSAD digestion, amplified using PCR primers (nt 1553–1949) that encompass both authentic cccDNA and dsl-cccDNA [8,13]. A key feature of dsl-cccDNA is the presence of various INDELs at the joint site, resulting from self-ligation events, presumably within the DR2–1 region. To identify tissue biopsies most likely containing dsl-cccDNA for further investigation, we first examined deletions within the region targeted by PSAD-cccDNA PCR assay (nt 1600–1840), which encompasses the DR2–1 region, using HBV-targeted NGS data from 56 liver biopsies, as described below.

**Fig 2:**
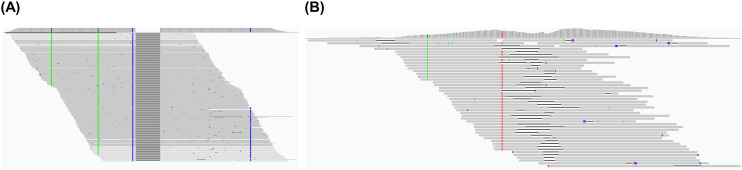
Two major deletion patterns identified in the DR2-1 (nt 1600–1840) region of the CHB tissue biopsies by HBV-targeted NGS assay. (A) An example of a 22-nt deletion at nt 1755–1776 identified in Pt 5 with 3,356 supporting reads (SRs). (B) An example of various lengths of deletions with 1 UMI-consolidated SR across nt 1684–1957. Blue, green, and red indicate SNPs. Note: Reads in IGV were condensed and may not show all supporting reads.

First, two distinct deletion patterns were observed using Integrative Genomics Viewer (IGV). The first pattern, illustrated in Fig 2A, shows deletions supported by many supporting reads (SRs). For example, a 22-nt deletion (nt 1755–1776) was supported by 3,356 SRs of 39,710 total HBV reads in Pt 5, indicating that thousands of double-stranded HBV DNA templates in ~100 ng of tissue DNA contained this deletion. The second pattern, illustrated in Fig 2B from Pt 4, shows many deletions each supported by a single unique molecular identifier (UMI)-consolidated SR, suggesting that each deletion originated from an individual DNA template. We reasoned that the second pattern is more likely derived from dsl-cccDNA, as each self-ligation represents an independent event, resulting in unique species of INDELs.

Next, we selected samples containing more than five distinct deletions in the DR2–1 region. To ensure that each deletion likely originated from an individual self-ligation event, we required that each deletion be supported by less than five UMI-consolidated SRs. Although multiple self-ligation events could theoretically produce the same deletion, or an iDNA carrying the deletion could expand through cell proliferation, we considered it highly unlikely for five independent self-ligation events to generate identical deletions. Therefore, less than five SRs was used as the criterion for self-ligation. Based on these two criteria, thirty-two samples, including two negative samples, as listed in column J of S1 Table, were selected from the HBV-targeted NGS dataset for PSAD-cccDNA PCR NGS study.

To prepare for NGS library construction, the 32 PCR products from PSAD-cccDNA PCR assay were first analyzed for size distribution by capillary electrophoresis. Notably, a significant proportion of the PCR products were shorter than the expected size of 397 bp (nt 1553–1949), as observed in all 32 samples. Examples from Pt 2, Pt 22, and Pt 41, highlighted by arrows in S1A Fig, illustrate this finding. To further investigate the composition of the PCR products, a pilot NGS was performed on the PCR products of the first five patients, Pt 2, Pt 16, Pt 17, Pt 22, and Pt 41. The results are summarized in the table inserted in S1B Fig. Although over one million NGS reads were obtained, over 99.9% did not contain both cccDNA primer sequences, indicating that they were non-specific PCR products. The majority of these reads were mapped exclusively to human genomic sequences. We thus performed DNA size fractionation to remove fragments samller than 200 bp to enrich for specific HBV PCR products for sequencing. As a result, we obtained 72.2–93.6% of NGS reads containing both primer sequences specific for the cccDNA PCR product. The resulting sequencing data were analyzed for INDELs in the DR2–1 region (nt 1600–1840), followed by concordance analysis, as summarized in S1 Table. INDELs concordantly detected by these two independent NGS assays were used to identify dsl-cccDNA for further characterization.

### Distribution of concordant INDELs identified by two independent NGS assays

**Fig 3:**
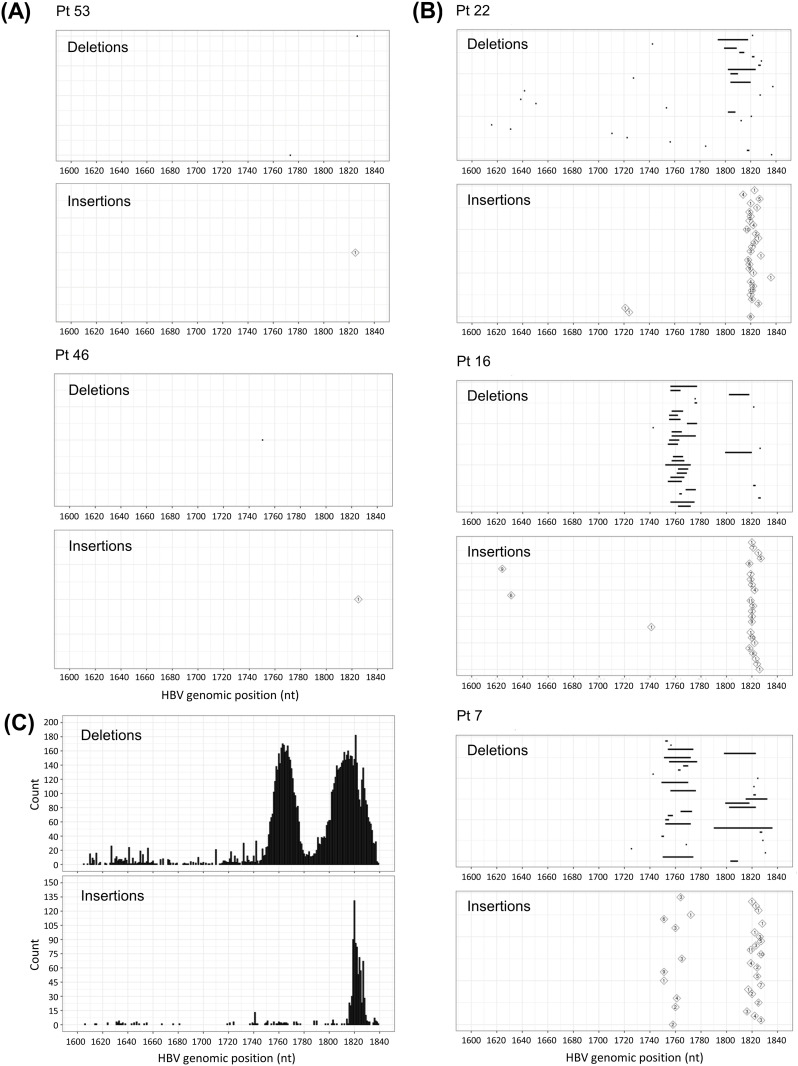
Distribution of concordant INDELs detected by PSAD-cccDNA PCR NGS. (A) INDELs detected in two negative controls. (B) Representative examples of the three observed INDEL distribution patterns. INDEL distribution of all 32 samlples, including the two negative controls, are detailed in S3 Fig. (C) Aggregate distribution of deletion (top) and insertion (bottom) positions among all concordant INDELs across 32 samples. Each unique INDEL was counted once per sample, regardless of the number of supporting reads. The counts represent the sum across all 32 samples.

INDELs at the joint region, presumably within the DR2–1 region, are a key characteristic of dsl-cccDNA. To investigate the features of these INDELs, we performed two analyses on the INDELs concordantly identified within nt 1600–1840 by both NGS assays. First, we characterized the concordant INDELs for their sizes, genomic positions, and frequencies using data from PSAD-cccDNA PCR NGS. The sizes and genomic positions of the 30 most abundant conrodant INDELs for each sample are illustrated in S2 Fig. Their frequencies were calculated and shown as sidebars. Two negative controls selected by HBV-targeted NGS, Pt 53 and Pt 46, defined by having fewer than five distinct INDELs, contained 2 and 3 qualified deletions (<5 SRs) in HBV-targeted NGS, and had 2 and 1 deletions in concordance between the two NGS assays, respectively (Fig 3A). Therefore, the two negative controls became positives in the concordance study when incorporating INDELs detected by a more sensitive PSAD-cccDNA PCR NGS. Among the 30 biopsies selected as positive candidates for dsl-cccDNA, three types of INDEL distribution patterns (Type I–III) were identified based on the primary clustering of their genomic locations, as individually illustrated in S2 Fig and represented by three liver biopsies in Fig 3B. First, Pt 22, representing 9 biopsies and categorized as Type I, exhibited both insertions and deletions primarily clustered around nt 1820, near the DR1 site (nt 1824–1834). Second, Pt 16, representing 20 biopsies and categorized as Type II, showed deletions clustered at around nt 1760, with or without a second cluster around nt 1820, while insertions were predominantly centered at nt 1820. Third, Pt 7, the only biopsy classified as Type III, displayed both insertions and deletions centered at both nt 1760 and nt 1820 (S2 Fig). Collectively, deletions observed across all 30 biopsies formed two major clusters, whereas insertions were predominately concentrated around the DR1 site (nt 1820), as shown in Fig 3C.

### Discovery of a 22-nt deletion in the X coding region

**Fig 4:**
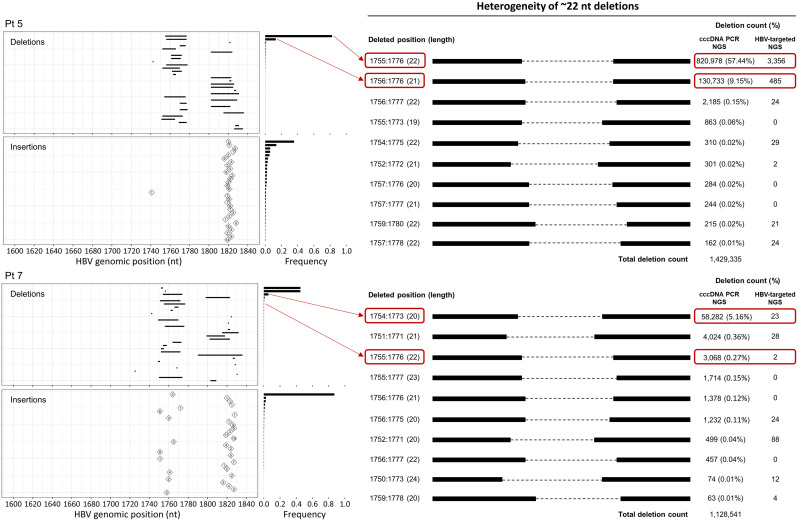
Heterogeneity of~22-nt deletions around nt 1760 in two representative samples, Pt 5 and Pt 7. The positions and lengths of deletions are indicated, along with the supporting reads (deletion count) and their proportions relative to all deletions identified within the nt 1600–1840 region. Data are shown from both HBV-targeted and PSAD-cccDNA PCR NGS assays.

By examining the distribution and frequencies of the 30 most frequent INDELs across all 30 dsl-cccDNA-positive samples (S2 Fig), we observed that in some patients, one or a few deletions accounted for more than 20% of all concordant deletions, such as a 22-nt deletion (nt 1755–1776) from Pt 5. As illustrated in Fig 4, this 22-nt deletion accounted for 86% of the concordant deletions and 57.4% of all deletions detected by PSAD-cccDNA PCR NGS in Pt 5. A total of 3,356 SRs were identified from HBV-targeted tissue NGS, suggesting the presence of at least thousands of HBV cccDNA molecules harboring this specific deletion. Notably, the second most frequent deletion in Pt 5 was also located within the same genomic region. This finding contradicts one of our dsl-cccDNA selection criteria, which deletions resulting from individual self-ligation events should be present in only a single species of dsl-cccDNA, unless identical deletions were created independently from multiple ligation events, which is expected to be rare and thus low in frequency. To investigate the prevalence of this 22-nt deletion, we examined its presence across samples. Surprisingly, this specific deletion was found in all 32 samples, with a higher abundance observed in HBeAg(-) patients (S3 Fig), as detected by the PSAD-cccDNA PCR NGS assay. For instance, in Pt 7, this 22-nt deletion accounted for 0.27% of all deletions detected by PSAD-cccDNA PCR NGS (Fig 4). Notably, in samples where more than 20% of deletions were clustered around the nt 1760 region, the data suggested that these deletions may not result from individual self-ligation events. Instead, they likely originated from amplification of cccDNA molecules already harboring this 22-nt deletion, possibly inherited from the rcDNA precursor through rcDNA recycling pathway or *de novo* infection.

To investigate the sources of the abundant deletions around nt 1760, we first examined if this highly abundant 22-nt deletion observed in Pt 5 had been previously reported. A BLAST search was conducted using the HBV DNA sequence from nt 1680–1798 with the nt 1755–1776 deletion as a query sequence. The top 100 matching sequences from GenBank were retrieved and analyzed, as summarized in S2 Table. While this specific 22-nt deletion has not been previously reported in GenBank or literature to our knowledge, 78% of the top 100 HBV DNA sequences shared over 90% identity with the query sequence, suggesting that deletions in this region may be at high prevalence. Several ~20-nt heterogenous deletions, such as nt 1753–1772 or 1758–1777, that can affect the X gene, basic core promter (BCP), and enhancer II (ENII) regions have been previously reported in CHB patients [14], and were also identified in our study population (S3 Table). Next, we analyzed the size composition of deletions within the 32-nt region of nt 1750–1781. As shown in S4 Fig, deletions in this region were identified in 49 of the 56 samples (88%) using HBV-targeted NGS. 1-nt deletions were the most common ones, observed in 47 of the 49 samples (96%). Larger deletions ranging from 18–23 nt were found in 14 patients (29%).

### Validation of the newly discovered 22-nt deletion (nt 1755–1776) by PCR cloning and Sanger sequencing

To validate the presence of this newly discovered 22-nt deletion (nt 1755–1776), we selected two samples, Pt 5 and Pt 7, for PCR cloning and Sanger Sequencing. This deletion accounted for 57.4% and 0.27% of all deletions identified by PSAD-cccDNA PCR NGS in Pt 5 and Pt 7, respectively. Two sets of primers were designed for validation, as illustrated in S5 Fig and detailed in Materials and Methods. In Pt 5, the deletion was detected in 3 of 4 (75%) clones using primers for both wild-type and deleted templates, and 1 of 1 (100%) clone using deletion-specific primers. In Pt 7, the deletion was identified in 2 of 10 (20%) clones using primers for both wild-type and deleted templates (S5B and S5C Fig). These results confirmed the presence of the 22-nt deletion, which was detected in all 32 CHB patients analyzed in this study.

### Size distribution of INDELs occurring in nt 1800–1840

To further characterize the INDELs of dsl-cccDNA, we analyzed the size of each insertion and deletion across all 32 PSAD-cccDNA PCR NGS samples within the nt 1800–1840 region. The INDEL size distribution for each biopsy was plotted in the S6 Fig and collectively summarized in Table 1. Consistent with previous findings from tissue culture and animal models [7–12], most INDELs of dsl-cccDNA were less than 10 nt. 81% of insertions were shorter than 10 nt, with the largest insertion measuring 111 nt. Similarly, 61.1% of deletions were under 10 nt, and the longest deletion was 87 nt. Overall, only about 1% of INDELs over nt 1800–1840 region exceeded 50 bp in length.

**Table 1:** Size distribution of INDELs detected in dsl-cccDNA over nt 1800–1840 in CHB patients.

Type	Length (nt)					
	1–10	11–20	21–30	31–40	41–50	50 + , Max (bp)
Insertion	80.8%	11.8%	3.2%	1.6%	1.4%	1.1%, 111
Deletion	61.6%	17.0%	11.4%	4.2%	4.8%	1.0%, 87

### Prevalence of dsl-cccDNA

**Fig 5:**
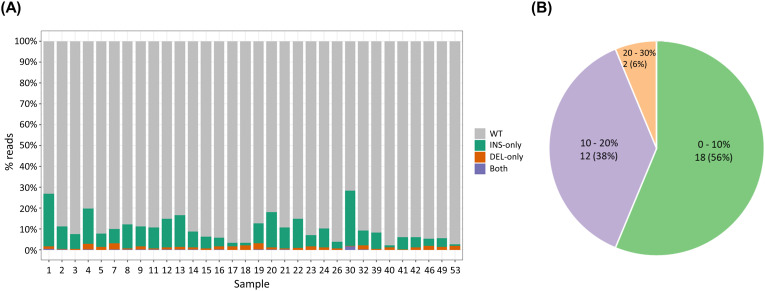
Abundance of intrahepatic dsl-cccDNA (INDELs in nt 1800–1840) in CHB patients, as determined by PSAD-cccDNA PCR NGS assay. (A) The composition of intrahepatic cccDNA (WT) and dsl-cccDNA (INDELs in nt 1800–1840) in each patient. (B) Summary of dsl-cccDNA proportion relative to total cccDNA in the 32 CHB patients.

As we concluded that deletions clustered in the nt 1760 region can be found in authentic cccDNA, we therefore refined our definition of dsl-cccDNA to include only those INDELs found at the DR1 end-joining site (nt 1800–1840). We then assessed the prevalence of dsl-cccDNA in our study cohort. INDELs at the DR1 end-joining site were detected in all 32 subjects, indicating a 100% prevalence of dsl-cccDNA among patients with detectable cccDNA. We further calculated the proportion of dsl-cccDNA relative to total cccDNA, as summarized in Fig 5. Although dsl-cccDNA was detectable in all patients, the majority of cccDNA (71.8–97.8%) lacked detectable insertions or deletions and were classified as authentic cccDNA (wild-type, WT), shown in grey. Among the detected INDELs, the templates containing insertions only (0.76–26.4%) were significantly (p < 0.001, Wilcoxon Signed-Rank test) more abundant than the templates containing deletions only (0.08–3.00%). Only a small fraction (0.0004–1.73%) of the templates contained both deletions and insertions. Among the 32 CHB patients with detectable cccDNA, 18 (56%) had less than 10% of dsl-cccDNA. Only 2 (6%) had over 20% of their cccDNA identified as dsl-cccDNA, as summarized in Fig 5B.

To corroborate our findings from patient tissue biopsies in the concordance studies using two independent NGS assays, we performed INDEL analysis with the same approach on DNA isolated from three tissue culture models, HBV-infected PHH (HBV-PHH), HBV-infected HepG2-NTCP (HBV-HepG2-NTCP), and HepAD38 (tet-), as described in Matherials and Methods. As summarized in S7A Fig, both HBV-infected tissue culture models showed a similar INDEL distribution pattern, except for the absence of the 20–22 nt deletions observed in all tested patient liver biopsies (Fig 3C). Consitent with our tissue biopsy results (Fig 5) and a previous report from tissue culture [10], dsl-cccDNA accounted for 2–4% of total cccDNA in these three tissue culture models. This proportion of dsl-cccDNA and INDELs pattern in the DNA isolated from HepAD38 (tet-) tissue culture were further validated using two independent enzymatic approaches—T5 exonuclease and Exonuclease I/III—to remove non-cccDNA species (S7B Fig). Of interest, our analysis of a publicly available RNAseq dataset [15] from CHB patients identified INDELs at the DR1 end-joining site (nt 1800–1840) (S4 Table), suggesting that these transcripts were likely derived from dsl-cccDNA.

### Dsl-cccDNA is more abundant in HBeAg(+) CHB patients

**Fig 6:**
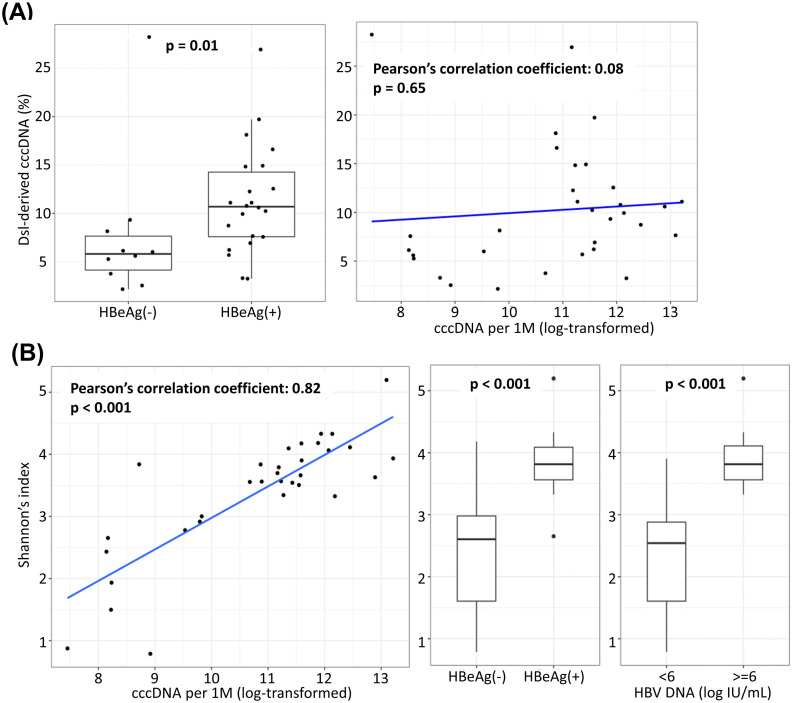
Association between dsl-cccDNA adunance and viral factors. (A) Comparison of the proportion of dsl-cccDNA in total cccDNA between HBeAg(+) and HBeAg(-) patients (left; p = 0.01, Wilcoxon Rank-Sum test), and correlation between dsl-cccDNA proportion and the cccDNA levels (right; p = 0.65, Pearson’s correlation test). (B) Relationships between the diversity of dsl-cccDNA and viral factors: cccDNA levels (left; p < 0.001, Pearson’s correlation test), HBeAg status (middle; p < 0.001, Wilcoxon Rank-Sum test), and serum viral load (right; p < 0.001, Wilcoxon Rank-Sum test).

As dsl-cccDNA is a by-product of HBV replication, it was of interest to determine if dsl-cccDNA is more abundant in HBeAg(+) samples, where the amount of dslDNA, as its precursor, should be higher. As shown in Fig 6A, the proportion of dsl-cccDNA in total cccDNA was significantly higher in the HBeAg(+) group compared to the HBeAg(-) group, with a mean 11.3% (3.2–26.9%) *vs.* 7.7% (2.2–28.2%) (p = 0.01). However, no significant correlation was obseved between the proportion of dsl-cccDNA and cccDNA levels (r = 0.08, p = 0.65).

Next, we determined the diversity of dsl-cccDNA species and its relationships with various viral factors, including cccDNA levels and other viral blood biomarkers. The diversity of dsl-cccDNA species was calculated using Shannon’s diversity index, as detailed in Materials and Methods. As shown in Fig 6B, dsl-cccDNA diversity showed a strong positive correlation with cccDNA levels (log-transformed; r = 0.82, p < 0.001), and was significantly associated with HBeAg status (p < 0.001) and serum viral loads (p < 0.001). The proportion of dsl-cccDNA, as well as the sizes and genomic locations of dsl-cccDNA deletions, were compared among HBV genotypes A–D. No significant difference was observed.

## Discussion

Although hepadnaviral dsl-cccDNA has been shown in cell culture and animal models (duck and woodchuck) for over two decades, this study is the first to detect and characterize dsl-cccDNA in liver tissue from CHB patients. By concordance analysis between two independent NGS assays—HBV-targeted NGS of 56 liver needle biopsies and PSAD-cccDNA PCR NGS of 32 of 56 samples—we defined and characterized dsl-cccDNA based on the genomic positions and sizes of INDELs around the DR1 end-joining site, centered at nt 1820. Notably, a recurrent 22-nt deletion (nt 1755–1776) in the X gene was discovered in the cccDNA of all 32 patients by PSAD-cccDNA PCR NGS assay (Fig 4 and S3 Table). Comparison of dsl-cccDNA abundance between the 22 HBeAg(+) and 10 HBeAg(-) patients revealed significantly higher proportion of dsl-cccDNA in the HBeAg(+) group (p = 0.01), independent of their cccDNA levels (Fig 6A). Furthermore, the diversity of dsl-cccDNA species strongly correlated with cccDNA levels (r = 0.82, p < 0.001), and was associated with HBeAg status (p < 0.001) and serum viral loads (p < 0.001) (Fig 6B).

Given that dsl-cccDNA is formed *via* the error-prone NHEJ machinery, different self-ligation events are expected to result in various joint sequences with different insertions or deletions. As a result, INDELs in dsl-cccDNA are typically supported by one to a few reads. In this study, we first identified samples likely to contain dsl-cccDNA by detecting the “dsl-cccDNA-like” INDELs at the DR1 end-joining site (nt 1800–1840), as illustrated in Fig 2B, using HBV-targeted NGS of 56 tissue biopsies from CHB patients. As outlined in Fig 1, we then selected 32 samples, including two negative controls, for PSAD-cccDNA PCR NGS analysis to identify and characterize dsl-cccDNA in infected livers through concordance analysis. The first round of PSAD-cccDNA PCR NGS of five samples yielded over 99.9% non-specific PCR products, likely due to non-specific amplification by short DNA fragments originating from incomplete digestion of linear DNA that served as primers, resulting in only 0.004–0.005% of NGS reads containing both HBV primers (S1 Fig).

Four key observations were documented in this study. **First**, we detected high prevalence of dsl-cccDNA in CHB patients in our study cohort. All 32 liver biopsies contained detectable dsl-cccDNA by PSAD-cccDNA PCR NGS assay, including the two negative controls selected by HBV-targeted NGS assay. This may not be surprising because self-ligation of dslDNA to form dsl-cccDNA could occur as frequently as integration, which involves recombination with host DNA and is known to be highly prevalent.

**Second,** consistent with previous reports from tissue culture and animal studies [9,10], we detected a heterogenous pattern of INDELs. None of the INDEL species at the DR1 end-joning site (nt 1800–1840) had more than 10 SRs, suggesting that dsl-cccDNA in CHB patients should be mostly defective for replication and, if functional, may only persist for a few generations. Interestingly, analysis of a publicly available RNAseq dataset from CHB patients [15] indicates that some of these dsl-cccDNA can serve as templates for RNA transcription (S4 Table). Collectively, the dsl-cccDNA pool could represent a mixture of cccDNA converted from infecting viruses carrying dslDNA genomes, progeny dsl-cccDNA replenished by illegitimate replication, and those converted from dslDNA byproduct during legitimate replication supported by authentic cccDNA. This pool of INDELs is mediated by error-prone NHEJ DNA repair pathway, giving rise to an extensive and heterogenous pattern of INDELs at the DR1 region.

**Third,** the majority of dsl-cccDNA INDELs were shorter than 10 nt, with the largest detected deletion and insertion spanning 111 bp and 87 bp, respectively (Table 1 and S6 Fig). Deletions larger than 50 bp comprised a mere 1% of the total, while an overwhelming 90% of deletions were limited to 30 bp or shorter.

**Fourth**, we discovered a 22-nt deletion (nt 1755–1776) potentially impacting the X/BCP/ENII regions, validated by PCR cloning and Sanger sequencing of tissue biopsy DNA (S5 Fig). This deletion was universally present in all 32 CHB subjects studied. BLAST analysis using both the wild-type (119-nt) and deleted (97-nt) sequences against GenBank revealed 85% – 100% of identity with the top 100 most homologous complete genomes (S2 Table). Although we did not find an identical 22-nt deletion in GenBank, 78 of these 100 sequences shared more than 90% identity with the deleted sequence, suggesting this 22-nt deletion occurs in a similar region to previously reported heterogeneous 20-nt deletions (nt 1753–1777) associated with dysfunctional X-protein, as part of HBV quasi-species [14]. Moreover, this study [14] also found INDEL mutation frequency was negatively correlated with HBeAg levels in CHB patients prior to antiviral treatment. Consistent with these findings, the 22-nt deletion in our study was at frequencies ranging from <1% to 46% of total cccDNA by PSAD-cccDNA PCR NGS, with a higher abundance observed in HBeAg(-) patients (S3 Table and S3 Fig). Interestingly, we did not detect the 20–22 nt deletions in any of the three tissue culture infection models tested (S7 Fig), suggesting that the deletions observed around nt 1760 may be specific to chronic infection in patients and accumulate over time. Further study are needed to validate this hypothesis.

Although this study presents the first comprehensive characterization of dsl-cccDNA and its prevalence among CHB patients, several limitations and caveats should be acknowledged. **First**, all the dsl-cccDNA reported herein were limited to those containing the PCR amplicon region spanning nt 1553–1949. It is plausible that mutants or truncated forms of dsl-cccDNA lacking the PCR amplicon region or primer-binding sites may exist in infected livers. Moreover, the dslDNA may form oligomeric structures, either linear or circular, where monomeric dslDNA units are joined near their ends in random orientation [12,16]. These junctions are likely to harbor INDELs generated by NHEJ. While the dslDNA-derived linear concatemers will be efficiently removed in our heat/PSAD-cccDNA assay or by T5 or Exonuclease I/III digestion, the even rare dslDNA-dervied circular concatemers may still be scored as a dsl-cccDNA and included in the analysis. Encouragingly, the existence and the INDELs pattern of dsl-cccDNA in HepAD38 (tet-) tissue culture system characterized by our heat/PSAD-cccDNA PCR NGS assay were validated by using T5 and Exonuclease I/III cccDNA PCR NGS assays. Further investigation is warranted to determine the presence and significance of additional forms derived from dslDNA. **Second**, potential contamination by rcDNA may occur in our assay due to incomplete removal of rcDNA by heat-denaturation and PSAD treatment. If residul rcDNA originating from wild-type cccDNA were PCR-amplified, it would lack INDELs but could potentially lead to an underestimation of the fraction of dsl-cccDNA. On the other hand, although dsl-cccDNA predominately supports dslDNA replication, it remains possible that certain dslDNA-derived cccDNA species with miminal INDELs may produce rcDNA-like molecules [9,10], which, if not fully eliminated by heat and PSAD treatment, may be detected in our assay. However, such occurances are expected to be rare and would still reflect the INDELs of the original dsl-cccDNA, thus minimally affecting the overall profiling of dsl-cccDNA. **Third**, PCR amplification may introduce errors or mutations in the assay. Nonetheless, the INDELs introduced by PCR should be randomly distributed and not exhibit the DR1 site-focused INDEL pattern of dsl-cccDNA. **Lastly**, some “dsl-cccDNA” species with minimal INDELs may be mutant forms of rcDNA-derived cccDNA, given that cccDNA itself likely undergoes a dynamic process of DNA damage, editing, and repair in hepatocytes with a complex interplay of intrinsic and extrinsic factors over time. The plausible heterogeneity of cccDNA population in CHB patients await further investigation.

Since dsl-cccDNA is formed through self-ligation of dslDNA, byproducts of viral replication, we hypothesized that more active viral replication would result in more copies (quantity) and more variants (diversity) of dsl-cccDNA. In line with our hypothesis, both the quantity and diversity of dsl-cccDNA were significantly higher in patients with active viral replication, as indicated by HBeAg status (Fig 6A and 6B) and serum viral loads (Fig 6B). It is worth noting that, similar to integrated HBV DNA [17], the majority of dsl-cccDNA retain HBV surface antigen promoters and ORFs, and may be competent for HBsAg expression, provided the polyadenylation signal downstream of DR1 remains intact and functional. However, the alteration of HBeAg and HBx coding capacity due to ORF disruption by INDELs, especially the latter, may require the presence of authentic cccDNA in the same cell to supply HBx *in trans* to activate dsl-cccDNA episome transcription. Chronic hepatitis in humans is known to be associated with the progressive accumulation of hepatocytes that exhibit low or undetectable levels of viral antigen expression or abnormal expression patterns. We suggest that this pleomorphic viral expression may be linked to the presence of hepatocytes that express defective cccDNA molecules such as dsl-cccDNA. More studies are needed to further investigate the role and regulation of intrahepatic dsl-cccDNA in CHB pathogenesis and its potential contribution to hepatocarcinogenesis.

## Materials and methods

### Ethics statement

Patient tissue specimens were collected through Hepatitis B Research Network (HBRN) Adult Cohort Study (clinical trial no. NCT01263587). The Adult Cohort Study protocols were approved by the Institutional Review Boards of all participating institutions, with each participant provided written informed consent. The full names of the Institutional Review Boards are detailed at ClinicalTrials.gov (https://clinicaltrials.gov/study/NCT01263587?term=NCT01263587&rank=1#contacts-and-locations).

### Study subjects

HBRN is a research network of 28 clinical sites across the U.S. and Canada, funded by the National Institutes of Health. It was initiated to study the natural history of CHB and to conduct clinical trials in both children and adult populations. The Adult Cohort Study enrolled HBsAg(+) subjects ≥18 years old from 21 locations between 2012 and 2017, who were not receiving antiviral therapy at the time of enrollment. For the present study, 56 [24 HBeAg(+), 32 HBeAg(-)] participants who were not on antiviral therapy and had available liver tissues within 24 weeks of their clinical and virological assessments were included. The clinical information is summarized in S5 Table.

### HBV-targeted NGS and sequencing analysis

Total DNA previously isolated from the frozen liver biopsies was used for HBV-targeted NGS. Approximately 100 ng of DNA was sonicated to 100–250 bp fragments and prepared into sequencing libraries using 12 PCR cycles with the xGen cfDNA & FFPE DNA library prep kit (Cat# 10010207, Integrated DNA Technologies, Coralville, Iowa), which includes unique molecular identifiers (UMIs), following the manufacturer’s instructions. Library DNA was then subjected to the JBS HBV-targeted NGS assay [18] (JBS Science Inc, Doylestown, PA). The captured library DNA was pooled and sequenced on Illumina MiniSeq platform with 2 × 151 bp paired-end settings. Sequencing reads were processed and analyzed using the JBS *Advanced ChimericSeq* pipeline (JBS Science). Briefly, sequences that passed pre-processing were aligned to a combined reference consisting of the human genome (GRCh38.p13) and 42 HBV genomic sequences [19] by BWA-MEM [20]. Consensus sequences were generated for each UMI using fgbio CallMolecularConsensusReads [21] to correct potential PCR and sequencing errors. HBV-specific reads were then extracted through an additional alignment to the HBV reference sequences.

### Identification and characterization of dsl-cccDNA by HBV-targeted NGS and PSAD-cccDNA PCR NGS assays

As outlined in Fig 1B, 56 liver biopsies were initially analyzed for INDELs in the DR2–1 region (nt 1600–1840). Given that INDELs arising from different self-ligation events can vary, and that INDELs caused by Illumina NGS sequencing errors with UMI incoporated are rare [22], all detected INDELs were considered in the data analysis. Deletions were further characterized based on the number of UMI-consolidated supporting reads (SR). Sequences of interest were visually examined with Integrative Genomics Viewer [23] to assess the likelihood that the deletions originated from dsl-cccDNA (dsl-cccDNA-positive candidates). A subset of 32 samples were selected for PSAD-cccDNA PCR NGS to perform an INDEL concordance analysis.

### Intrahepatic HBV cccDNA quantitation and preparation of PCR products

Approximately 0.5 mm-long segments of 16-gauge needle liver biopsy samples stored in RNA*later* stabilization solution (Invitrogen, #AM7020) were transferred to β-mercaptoethanol-supplemented RLT lysis buffer (Qiagen, #79216) and broken down in PowerBead Tubes, filled with Ceramic 1.5 mm beads (Qiagen, #13113–50) using Bead Mill 24 Homogenizer (Fisherbrand, #15-340-163). Next, total DNA and RNA were isolated using AllPrep DNA/RNA Micro Kit (Qiagen, #80284), followed by quantitative and qualitative analyses on NanoDrop One (Thermofisher Scientific). For HBV cccDNA analysis, a portion of total DNA prep was heat denatured and digested by plasmid-safe ATP-dependent DNase (PSAD) (Bioresearch Technologies, #E3101K) to remove non-circular DNA species, followed by cccDNA-specific qPCR quantification, as described previously [8,13,24–28]. Note, a pre-heating step that denatures rcDNA into single-stranded DNA was introduced prior to PCR, which allowed an efficient degradation of the denatured rcDNA by PSAD while leaving cccDNA as the only detectable DNA template for qPCR to ensure the specificity of the assay [8]. Mitochondrial DNA COX3 gene qPCR was used for normalization of cccDNA, and the copy number of the host gene human hemoglobin subunit β (HBB) quantified by qPCR was used to calculate the cccDNA copy number per million cells. The qPCR primers and probes are listed in S6 Table. PCR products from PSAD-cccDNA PCR assay were purified by QIAquick PCR Purification Kit (Qiagen, #28106) prior to sequencing.

### Preparation of cccDNA PCR products from cell culture samples

HepAD38 and HepG2-NTCP cells were maintained as previously described [29,30]. Freshly isolated PHH cells were obtained from the Human Liver Tissue and Hepatocyte Research Resource (HLTHRR, funded by NIDDK project #R24DK139775) at The Pittsburgh Liver Research Center (PLRC, funded by NIDDK grant #P30DK120531), University of Pittsburgh, and cultured as previously described [25,26]. To induce HBV replication and cccDNA formation in HepAD38 cells, tetracycline (tet) was withdrawn from the culture medium, and cells were cultured for 18 days before harvest. HBV infection of HepG2-NTCP (HBV-HepG2-NTCP) and PHH (HBV-PHH) cells were conducted according to our publications [25,31,32], and the cells were infected for 6 days before harvest. The harvested cells were subjected to Hirt DNA extraction, followed by heat-denaturation and PSAD-treatment, T5 exonuclease (NEB, #M0663) digestion, or exonuclease I and III (Exo I/III) (NEB, #M0293 and #M0206) digestion, as previously described [8,33–35]. The treated DNA samples were subsequently column-purified and amplified by PCR using cccDNA-specific primers (S6 Table) and Phusion High-Fidelity DNA Polymerase (Thermo Fisher Scientific, #F530S). The PCR products were further purified by QIAquick PCR Purification Kit (Qiagen, #28106) prior to sequencing.

### cccDNA PCR NGS and INDELs analysis

Purified PCR products from PSAD-, T5-, or Exo I/III cccDNA PCR assays were subjected to library preparation using xGen DNA Lib Prep MC UNI 96rxn (Integrated DNA Technologies, #10009820) with UMI. For cell culture samples, single-end sequencing (1 × 291 bp) was performed on Illumina MiniSeq. Reads were trimmed using fastq [36,37], followed by sequence alignment using BWA-MEM. UMI-assisted deduplication was done using picard [38]. Sequences carrying either side of PCR primers were identified as PCR targtes and extracted for downstream analysis.

For patient samples, paired-end sequencing (2 × 250 bp) was performed by MedGenome (MedGenome Inc., Delaware, USA) on Illumina NovaSeq platform. Paired reads were trimmed and merged using fastp [36,37] to assemble full-length PCR products. The merged reads were then aligned to the HBV references using BWA-MEM and UMI-deduplicated using picard [38]. Sequences containing both PCR primer sequences were identified as PCR targets and extracted for downstream analysis. To to minimize the impacts of PCR artifacts, INDEL concordance analysis was performed.

INDELs concordantly detected by both NGS assays were identified and defined as originating from dsl-cccDNA. The INDEL patterns of dsl-cccDNA were characterized based on these concordant INDELs. INDELs detected in the nt 1800–1840 region by PSAD-cccDNA PCR NGS assay were further analyzed for INDEL size distribution, prevelance, proportion, and diversity. Shannon’s diversity index was applied to calculate INDEL species diversity based on frequency distribution of each sample. Each unique combination of INDEL positions within an individual NGS read was defined as an INDEL species. The frequency of each INDEL species were calculated per sample, such that the total frequency of all INDEL species in a given sample summed to 100%.

### Validation of a 22-nt HBV deletion by PCR cloning and Sanger sequencing

The HBV sequence containing a 22-nt deletion (nt 1755–1776) identified in Pt 5 and Pt 7 was analyzed for similarity to previously reported HBV sequences in GenBank. A reference sequence from genotype C (accession no. GQ377617.1) and the region of nt 1680–1798, with and without the 22-nt deletion, was used as the query for BLAST against NCBI nucleotide (nr/nt) database (taxid: 10407). Only hits with 100% query coverage and deposited as complete genomes were included in the comparison.

To validate the presence of the 22-nt deletion, two primer sets were designed based on the consensus sequences obtained from Pt 5 and Pt 7: one forward primer (nt 1680–1696)—ATGTCAACGACCGACCT—and two reverse primers—reverse 1 (nt 1798–1779), CTAATACAAAGACCTTTAACCT, and reverse 2, a deletion-specific primer (nt 1795–1753) AATTTATGCCTACAGCCTCAA, which span the deleted region (nt 1776–1755), as illustrated in S5A Fig. DNA isolated from two specimens (Pt 5 and Pt 7) was used to generate PCR products using Phusion High-Fidelity DNA Polymerase (Thermo Fisher Scientific, Waltham, MA, #F530S). The dA overhangs were added using native *Taq*DNA Polymerase (Thermo Fisher Scientific, #EP0282) or LA *Taq* DNA Polymerase (TaKaRa, San Jose, CA, #R002M). Haplotypes-containing fragments were ligated with a plasmid backbone using pGEM-T Easy Vector System (Promega, Madison, WI, #A137A) and sequenced via pUC/M13 forward and reversed sequencing primers. Alignment was performed using MEGA 7.0.26 software [39].

### Serum/plasma virological parameters

Serologies were assessed using sera collected on the same day as the biopsy. For participants missing quantitative HBsAg measurement on the biopsy day (as HBsAg was measured only once every 48 weeks per the study protocol), the nearest available HBsAg value within 48 weeks of the biopsy was used.

Quantitative HBV DNA (viral load) and qHBsAg were tested centrally by University of Washington. HBV DNA levels were determined using a real-time PCR assay (COBAS Ampliprep/COBAS TaqMan HBV Test, v2.0; Roche Molecular Diagnostics) with a lower limit of detection (LLOD) of 10 IU/mL and lower limit of quantification (LLOQ) of 20 IU/mL. Quantitative HBsAg was tested using the Roche Diagnostics Elecsys platform with LLOD of 0.05 IU/mL.

Quantitative HBV HBcrAg was tested centrally by Abbott Diagnostics. Serum HBcrAg concentrations were measured using a chemiluminescence enzyme immunoassay (Lumipulse G HBcrAg assay by Fujirebio Europe). The assay has a linear measurement range of 3.0 log_10_ to 6.8 log_10_ U/ml, with 3 log_10_ U/ml being the detection limit. Dilution was not performed for samples with concentration >6.8 log_10_ U/ml.

### Statistical analysis

Statistical analysis and data visualization were carried out on R Studio with R version 4.2.1 [40]. Wilcoxon Rank-Sum test (R function “wilcox.test”) was used to compare the proportion and diversity of dsl-cccDNA between HBeAg status [HBeAg(+) and HBeAg(-)] and between serum viral load (<6 log IU/ml and ≥6 log IU/ml), as well as the dsl-cccDNA proportion and deletion sizes among genotypes. Permutaional multivariate analysis of variance (PERMANOVA) test (R function “adonis”) was used to compare the deletion patterns among genotypes. Pearson’s correlation test (R function “cor.test”) was used to assess the relationships between the proportion of dsl-cccDNA and cccDNA levels, as well as between dsl-cccDNA diversity and cccDNA levels. A *p*-value <0.05 was considered statistically significant.

## Supporting information

S1 Table Summary of data obtained from HBV-targeted NGS and PSAD-cccDNA PCR NGS assays. (XLSX)

S2 Table Confirming the nt 1755–1776 deletion using BLAST. (XLSX)

S3 Table Detection of ~22-nt deletions around nt 1760 by PSAD-cccDNA PCR NGS. (XLSX)

S4 Table Detection of dsl-cccDNA expression from serum HBV RNAseq data. (DOCX)

S5 Table Baseline demographics and clinical features. (DOCX)

S6 Table List of oligonucleotides used in the study. (DOCX)

S1 Fig (A) Non-specific short (50–200 bp) PCR products from PSAD-cccDNA PCR assay revealed by Tapestation capillary electrophoresis. The arrows pointed to the position of the anticipated full-length PCR product of 397 bp. The short PCR products in a range of 50–200 bp were also noted. (B) Summary of NGS sequencing analysis. Two-primer reads are HBV reads (reads mapped to HBV references) containing both primers regardless the entire sequences. PCR target are HBV reads that have two primers on each end with additional 30 nt anticipated HBV sequences after each primer sequences.
(TIF)

S2 Fig INDEL distributions and frequencies of up to 30 most frequent INDELs that are concordant in the two NGS assays. Data displayed here is PCR NGS based. INDEL distributions were categorized into 3 types, Type I (A-I), Type II (J-C1), and Type III (D1), as detailed in the Results and Fig 3. The number of concordant reads were noted on top of each panel, as well as the total number of insertions and deletions. The frequencies of INDELs relative to the total number of insertions or deletions were represented by the sidebars with the range of INDELs noted on the side for INDELs with frequencies >0.1 (10%). The black bars in the top panel indicate the span of each deletions, and the diamonds in the bottom panel indicate the position of insertions, with the numbers indicating the insertion lengths.
(PPTX)

S3 Fig Frequency of the 22-nt deletion (nt 1755–1776) in total cccDNA of HBeAg(+) and HBeAg(-) patients. Wilcoxon Rank-Sum test was used to compare the freuquency between the HBeAg status (p < 0.001). An outlier, Pt 5 in HBeAg(+) group, was removed from the chart.
(TIF)

S4 Fig Deletion length distribution in nt 1750–1781. Y-axis denotes the prevalence of each deletion length among the 49 samples that contain detectable deletions in this region.
(TIF)

S5 Fig (A) Primer design for 22-nt deletion validation. One forward and two reverse primers were picked to make two sets of primers and three expected products. The reverse primer could amplify both sequences in WT (110 bp) and with 22-nt deletion (97 bp), while the reverse deletion-specific primer can only amplify sequences with this deletion (94 bp). (B) Sanger sequencing results of PCR cloning of the three types of products. (C) Cloning sequence alignment at the 22-nt deletion site.
(TIF)

S6 Fig Cumulative frequencies of insertion and deletion lengths detected by PSAD-cccDNA PCR NGS over the nt 1800–1840 region. (TIF)

S7 Fig Detection and characterization of dsl-cccDNA in tissue culture samples. (A) Distribution of INDELs detected in the nt 1600–1840 region from two HBV-infected tissue cultures: HBV-PHH and HBV-HepG2-NTCP. (B) Distribution of INDELs detected in the nt 1600–1840 region of HepAD38 cell-derived cccDNA samples using three different clean-up methods: heat+PSAD, T5, and Exo I/III. (C) Proportion of dsl-cccDNA in the tissue culture samples.
(TIF)
