# Supplementary material for: Detection and characterization of Hepatitis B virus double-stranded linear DNA-derived covalently closed circular DNA in chronic hepatitis B patients
Source: PLoS Pathog. 2026 Feb 24;22(2):e1013999. doi: 10.1371/journal.ppat.1013999 (PMC12952642; doi:10.1371/journal.ppat.1013999)
Supplement: S4 Table — (DOCX) [file ppat.1013999.s004.docx]

**S4 Table. Detection of dsl-cccDNA expression from serum HBV RNAseq data [1].**

| Sample* | Total HBV transcript reads** | dsl-cccDNA transcript reads*** | % dsl-cccDNA |
| --- | --- | --- | --- |
| 27 | 5,648,144 | 18,537 | 0.33% |
| 34 | 5,211,296 | 23,857 | 0.46% |
| 40 | 5,418,306 | 23,519 | 0.43% |
| *Top three samples with the most HBV reads from the referenced study  **reads not deduplicated | | | |
| ***criteria for dsl-cccDNA: INDELs in the DR1 region (nt 1800 - 1840) | | | |

1. Zaiets I, Gunewardena S, Menne S, Weinman SA, Gudima SO. Sera of Individuals Chronically Infected with Hepatitis B Virus (HBV) Contain Diverse RNA Types Produced by HBV Replication or Derived from Integrated HBV DNA. Journal of Virology. 2023;97(3):e01950-22. doi: doi:10.1128/jvi.01950-22.
