# Supplementary material for: Detection and characterization of Hepatitis B virus double-stranded linear DNA-derived covalently closed circular DNA in chronic hepatitis B patients
Source: PLoS Pathog. 2026 Feb 24;22(2):e1013999. doi: 10.1371/journal.ppat.1013999 (PMC12952642; doi:10.1371/journal.ppat.1013999)
Supplement: S5 Table — (DOCX) [file ppat.1013999.s005.docx]

**S5 Table. Baseline demographics and clinical features.**

|  | **HBeAg(+)**  **n=24** | **HBeAg(-)**  **n=32** | **P value** |
| --- | --- | --- | --- |
| **Age (years)** | 42.6  (23–67) | 46.5  (22–69) | 0.09 |
| **Gender (M:F)** | 12:12 | 22:10 |  |
| **Race**  **Asian**  **White**  **Black**  **Other** | 17  4  2  1 | 25  3  3  1 |  |
| **ALT (ULN)** | 3.9  (0.8–11.4) | 2.9  (0.8–21) |  |
| **Serum HBV DNA (log_10_ IU/ml)** | 7.5  (3.2–9.2) | 4.5  (2.6–7.7) | <0.00001 |
| **Serum qHBsAg (log_10_ IU/ml)** | 4.25  (1.94–5.35) | 2.96  (0.5–4.15) | <0.00001 |
| **cccDNA (per 1M cells)** | 147,363  (3,532–545,888) | 24,900*  (723–173,123) |  |
| **HBV genotype**  **A**  **B**  **C**  **D** | 5  4  11  4 | 5  15  7  5 |  |

*: BLOD (n=3) was excluded in the calculation.
