# Supplementary material for: Detection and characterization of Hepatitis B virus double-stranded linear DNA-derived covalently closed circular DNA in chronic hepatitis B patients
Source: PLoS Pathog. 2026 Feb 24;22(2):e1013999. doi: 10.1371/journal.ppat.1013999 (PMC12952642; doi:10.1371/journal.ppat.1013999)
Supplement: S6 Table — (DOCX) [file ppat.1013999.s006.docx]

**S6 Table. List of oligonucleotides used in the study.**

| **Target** | **Primer and probe sequences (5’→3’)** | |
| --- | --- | --- |
| HBV cccDNA | Forward | GTCTGTGCCTTCTCATCTGC |
|  | Reverse | AGTAACTCCACAGWAGCTCCAAATT |
|  | Probe | FAM-TTCAAGCCTCCAAGCTGTGCCTTGGGTGGC-TAMRA |
| Cytochrome c oxidase subunit III (COX3, mitochondrial) | Forward | CCCTCTCGGCCCTCCTAATAACCTGC |
|  | Reverse | GCCTTCTCGTATAACATCGCGTCA |
| Hemoglobin subunit β (HBB) | TaqMan Gene Expression Assay (FAM), with exact sequences of the primers and probe undisclosed (ThermoFisher Scientific, #4351370, #Hs00758889_s1) | |
