# Supplementary figures and images for: Detection and characterization of Hepatitis B virus double-stranded linear DNA-derived covalently closed circular DNA in chronic hepatitis B patients
Source: PLoS Pathog. 2026 Feb 24;22(2):e1013999. doi: 10.1371/journal.ppat.1013999 (PMC12952642; doi:10.1371/journal.ppat.1013999)

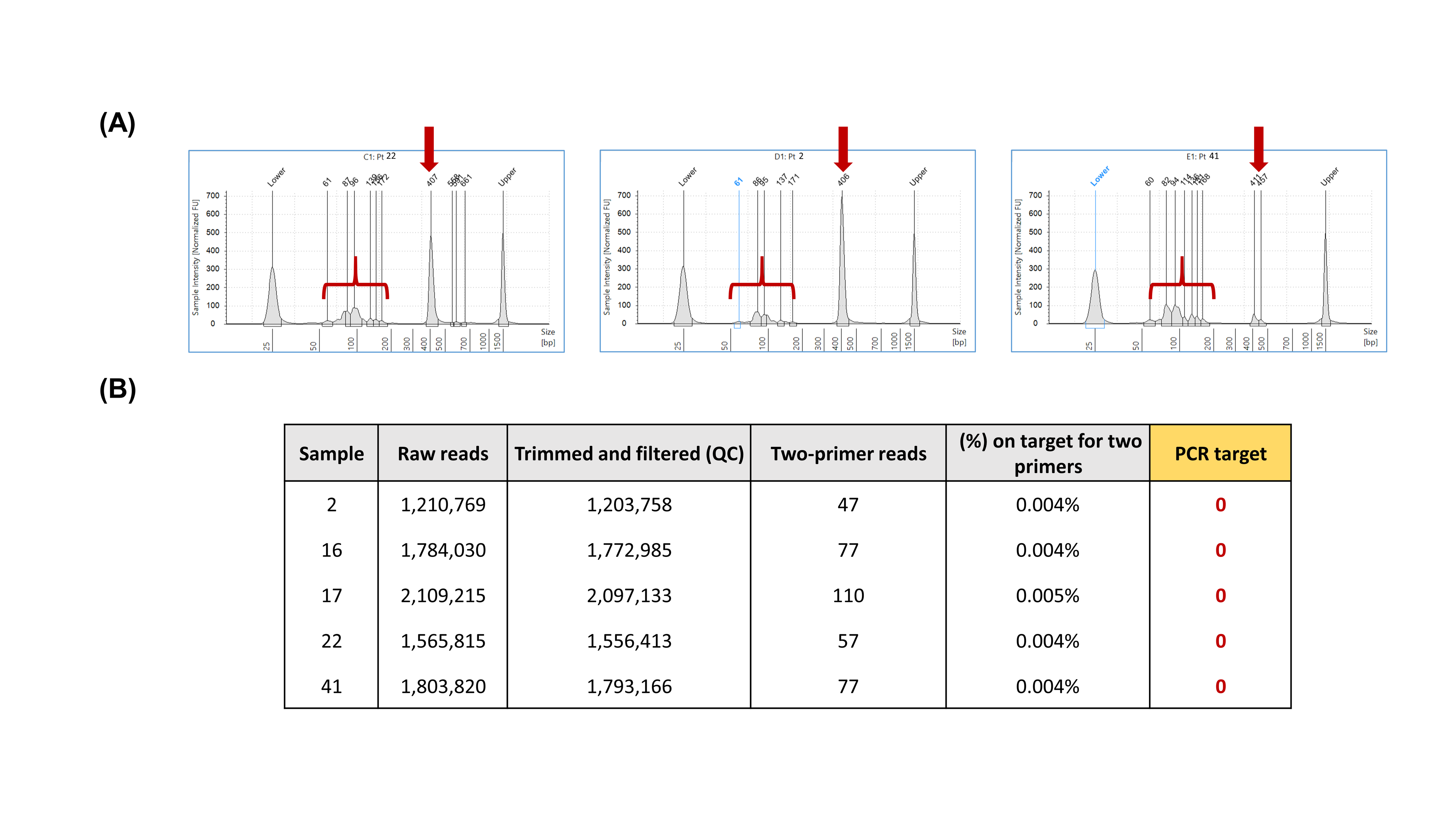

Supplement: S1 Fig — (A) Non-specific short (50–200 bp) PCR products from PSAD-cccDNA PCR assay revealed by Tapestation capillary electrophoresis. The arrows pointed to the position of the anticipated full-length PCR product of 397 bp. The short PCR products in a range of 50–200 bp were also noted. (B) Summary of NGS sequencing analysis. Two-primer reads are HBV reads (reads mapped to HBV references) containing both primers regardless the entire sequences. PCR target are HBV reads that have two primers on each end with additional 30 nt anticipated HBV sequences after each primer sequences. (TIF) [file ppat.1013999.s007.tif]

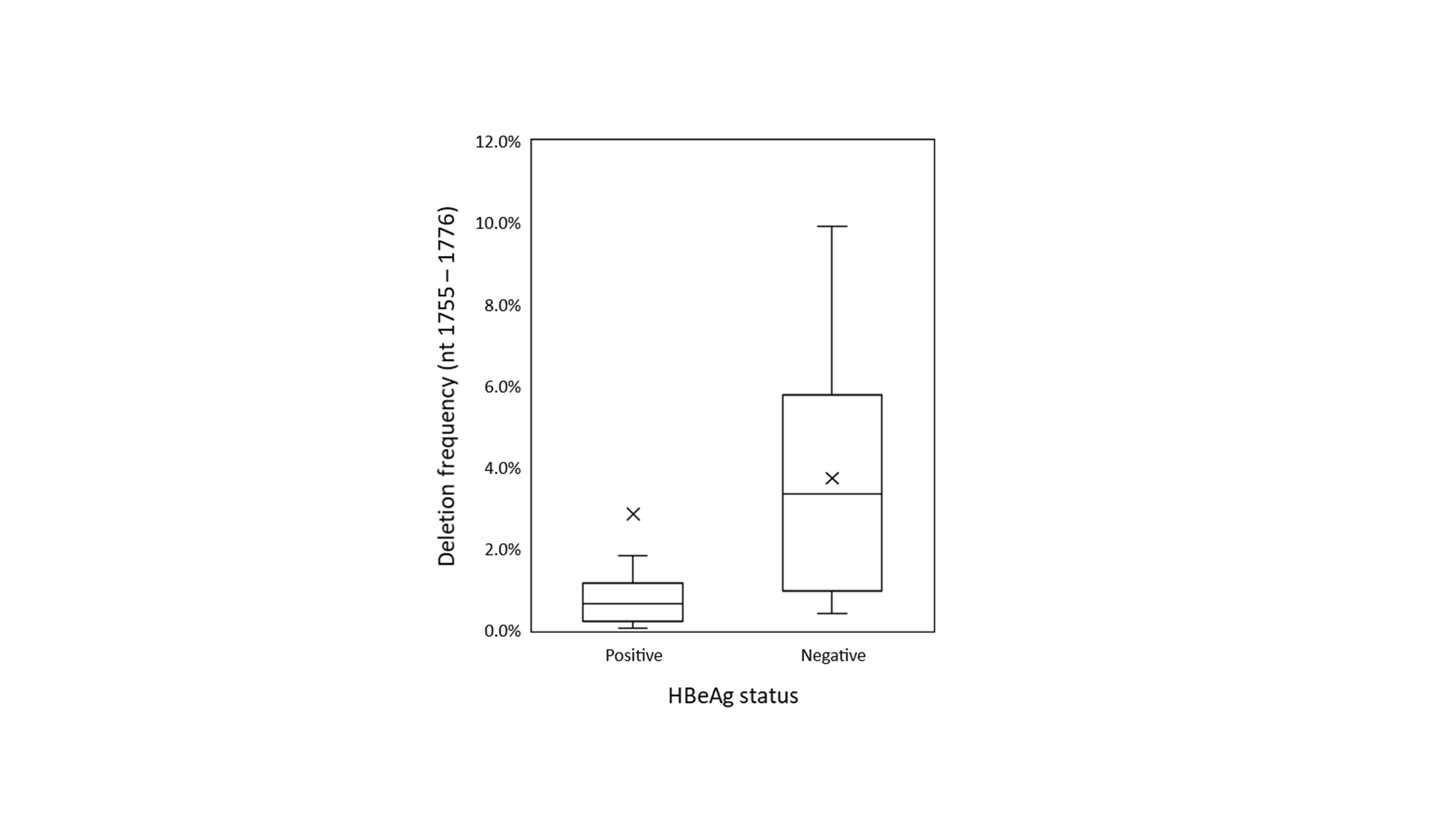

Supplement: S3 Fig — Wilcoxon Rank-Sum test was used to compare the freuquency between the HBeAg status (p < 0.001). An outlier, Pt 5 in HBeAg(+) group, was removed from the chart. (TIF) [file ppat.1013999.s009.tif]

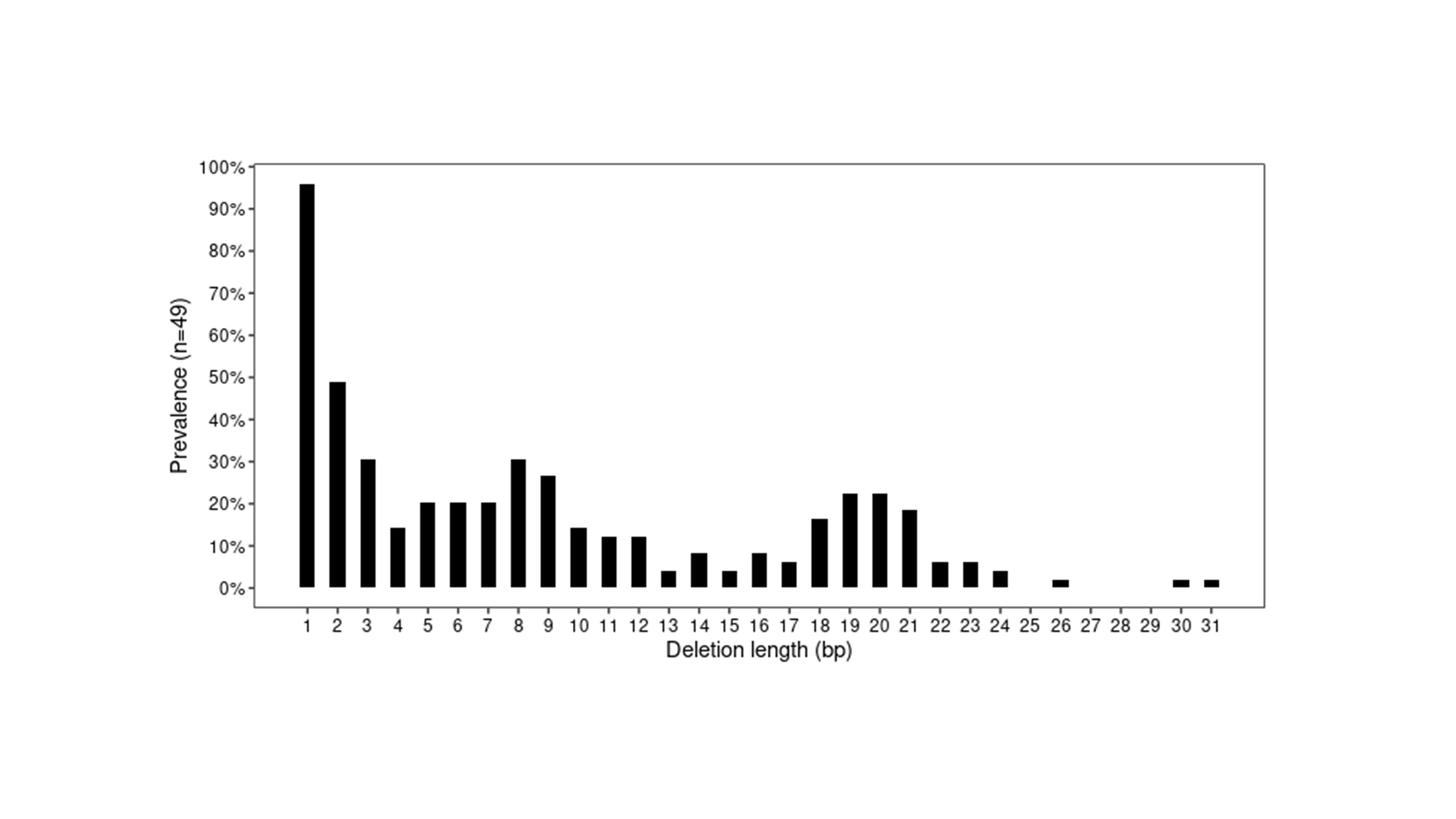

Supplement: S4 Fig — Y-axis denotes the prevalence of each deletion length among the 49 samples that contain detectable deletions in this region. (TIF) [file ppat.1013999.s010.tif]

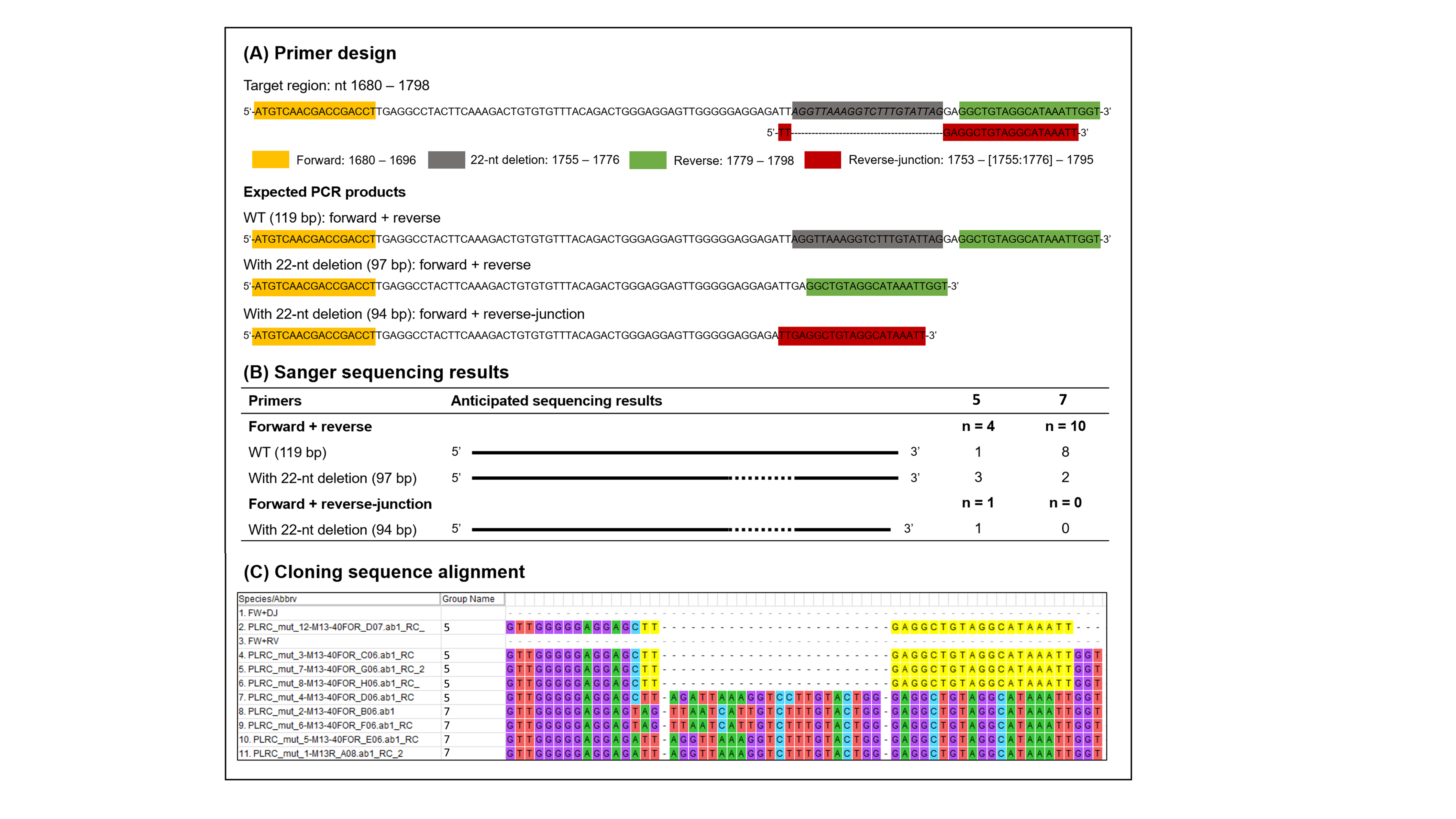

Supplement: S5 Fig — (A) Primer design for 22-nt deletion validation. One forward and two reverse primers were picked to make two sets of primers and three expected products. The reverse primer could amplify both sequences in WT (110 bp) and with 22-nt deletion (97 bp), while the reverse deletion-specific primer can only amplify sequences with this deletion (94 bp). (B) Sanger sequencing results of PCR cloning of the three types of products. (C) Cloning sequence alignment at the 22-nt deletion site. (TIF) [file ppat.1013999.s011.tif]

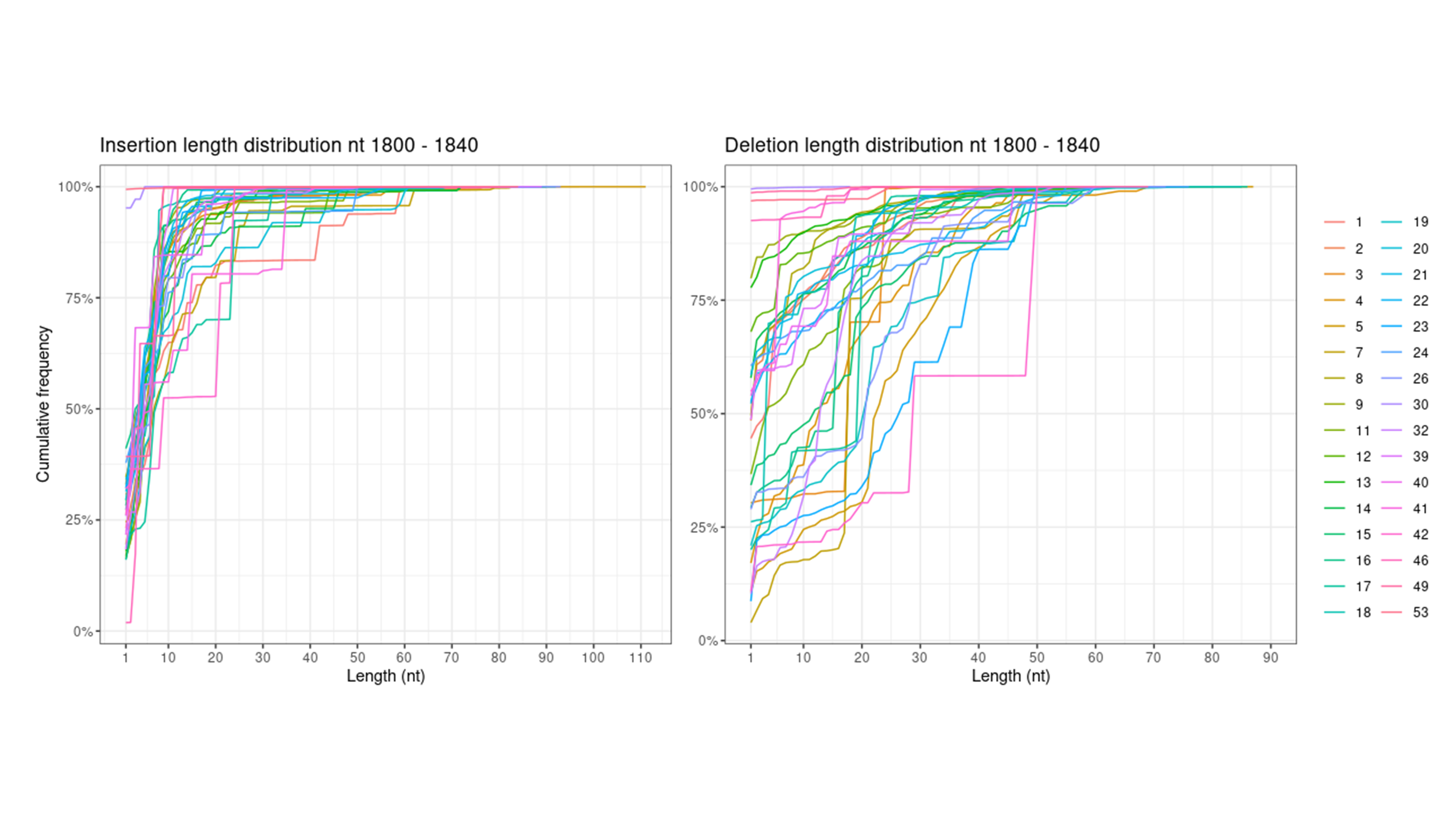

Supplement: S6 Fig — (TIF) [file ppat.1013999.s012.tif]

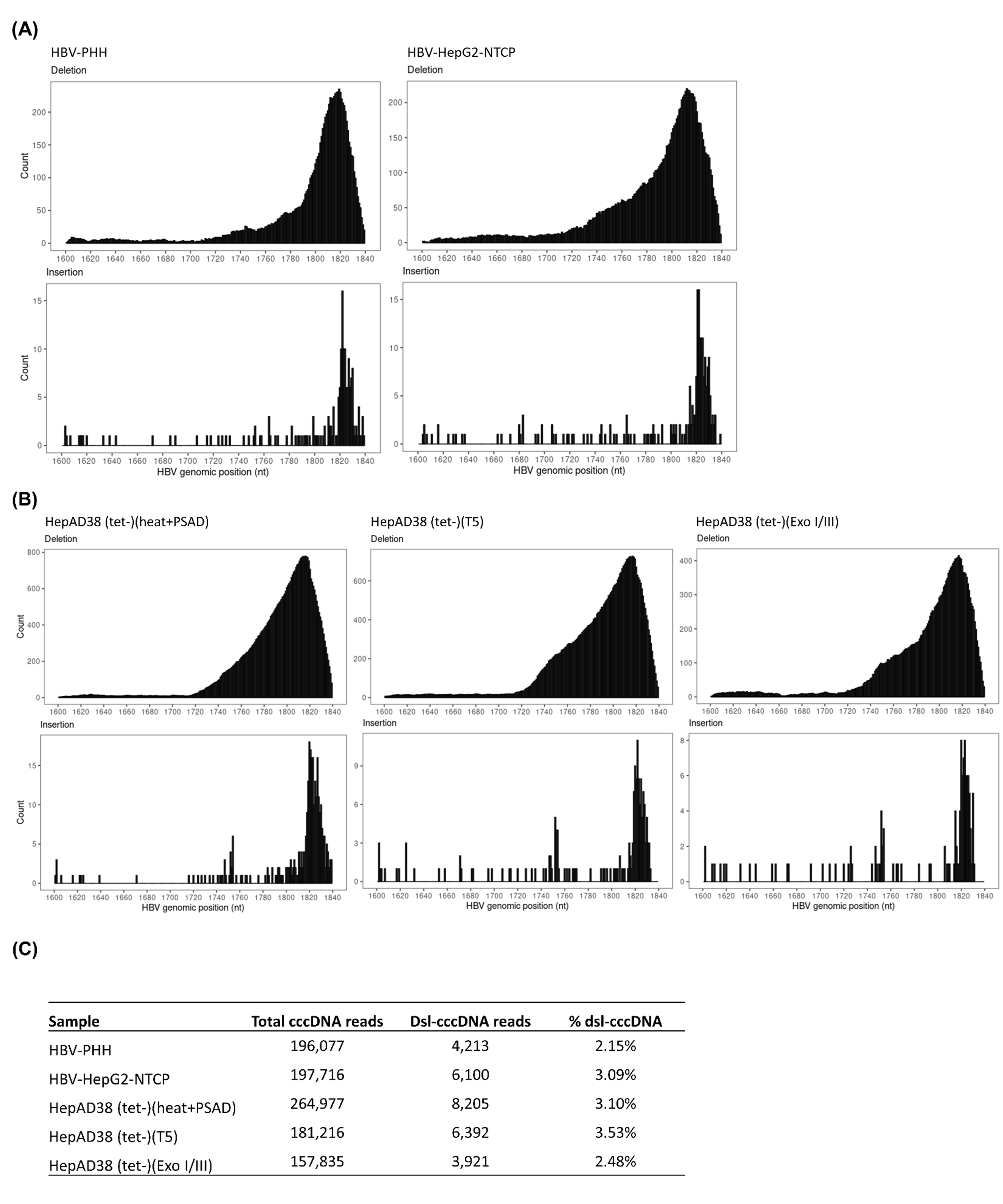

Supplement: S7 Fig — (A) Distribution of INDELs detected in the nt 1600–1840 region from two HBV-infected tissue cultures: HBV-PHH and HBV-HepG2-NTCP. (B) Distribution of INDELs detected in the nt 1600–1840 region of HepAD38 cell-derived cccDNA samples using three different clean-up methods: heat+PSAD, T5, and Exo I/III. (C) Proportion of dsl-cccDNA in the tissue culture samples. (TIF) [file ppat.1013999.s013.tif]
